# Case Report: Macrophage Activation Syndrome and Widespread Neuroimaging Abnormality in Childhood-Onset Systemic Lupus Erythematosus

**DOI:** 10.3389/fped.2021.767115

**Published:** 2021-12-14

**Authors:** Nana Shi, Xiangying Wang, Lixia Zou, Xinghui Yang, Qian Ma, Meiping Lu

**Affiliations:** ^1^Department of Hematology-Oncology, Children' Hospital, Zhejiang University School of Medicine, National Clinical Research Center for Child Health, Hangzhou, China; ^2^Hangzhou Children' Hospital, Hangzhou, China; ^3^Department of Rheumatology Immunology and Allergy, Children' Hospital, Zhejiang University School of Medicine, National Clinical Research Center for Child Health, Hangzhou, China; ^4^Department of Radiology, Children' Hospital, Zhejiang University School of Medicine, National Clinical Research Center for Child Health, Hangzhou, China

**Keywords:** macrophage activation syndrome, systemic lupus erythematosus, central nervous system, neuroimaging, pediatric

## Abstract

Macrophage activation syndrome (MAS) and widespread brain lesions are rare and severe complications of childhood-onset systemic lupus erythematosus (SLE). We report an 11-year-old girl who presented with recurrent rashes for half a year and fever for 2 weeks. Clinical and laboratory features at admission pointed to the diagnosis of SLE and SLE-associated MAS. Cerebral magnetic resonance imaging taken on day 4 after admission showed abnormal signals. Glucocorticoid therapy was started on day 5. Two days later, the patient appeared weak and ill, then the next day she exhibited dizziness, drowsiness, apathia, and dysarthria. High-dose methylprednisolone, cyclophosphamide, and intravenous immunoglobulin were used to treat the patient, and intrathecal dexamethasone was given. The patient was discharged on day 30 after admission and showed complete clinical resolution and improved magnetic resonance imaging resolution.

## Introduction

Systemic lupus erythematosus (SLE) is a clinically heterogeneous disease with variant manifestations and diagnosed on the basis of clinical and laboratory features. Macrophage activation syndrome (MAS) is a severe complication of rheumatic diseases and is currently classified among secondary forms of hemophagocytic lymphohistiocytosis (HLH). MAS manifests with fever, hepatosplenomegaly, central nervous system (CNS) dysfunction, hyperferritinemia pancytopenia, hepatic dysfunction, and coagulation abnormality. Compared with systemic juvenile idiopathic arthritis patients, SLE patients have significantly lower incidence of MAS, ranging from 0.9 to 9% (1–4). Widespread neuroimaging abnormality, which is a severe organ damage, was rarely reported in SLE-associated MAS. The present article reports a child with MAS and widespread neuroimaging abnormality as initial presentations at SLE diagnosis and describes the clinical course and management.

## Case Description

An 11-year-old girl was admitted to our hospital because of recurrent rashes for half a year and high fever for 2 weeks. The patient often complained of knee and instep pain in the past few years, and she had acute pancreatitis twice in the past year. She had no history or family history of autoimmune disease, cancer, or tuberculosis.

**Figure 1:**
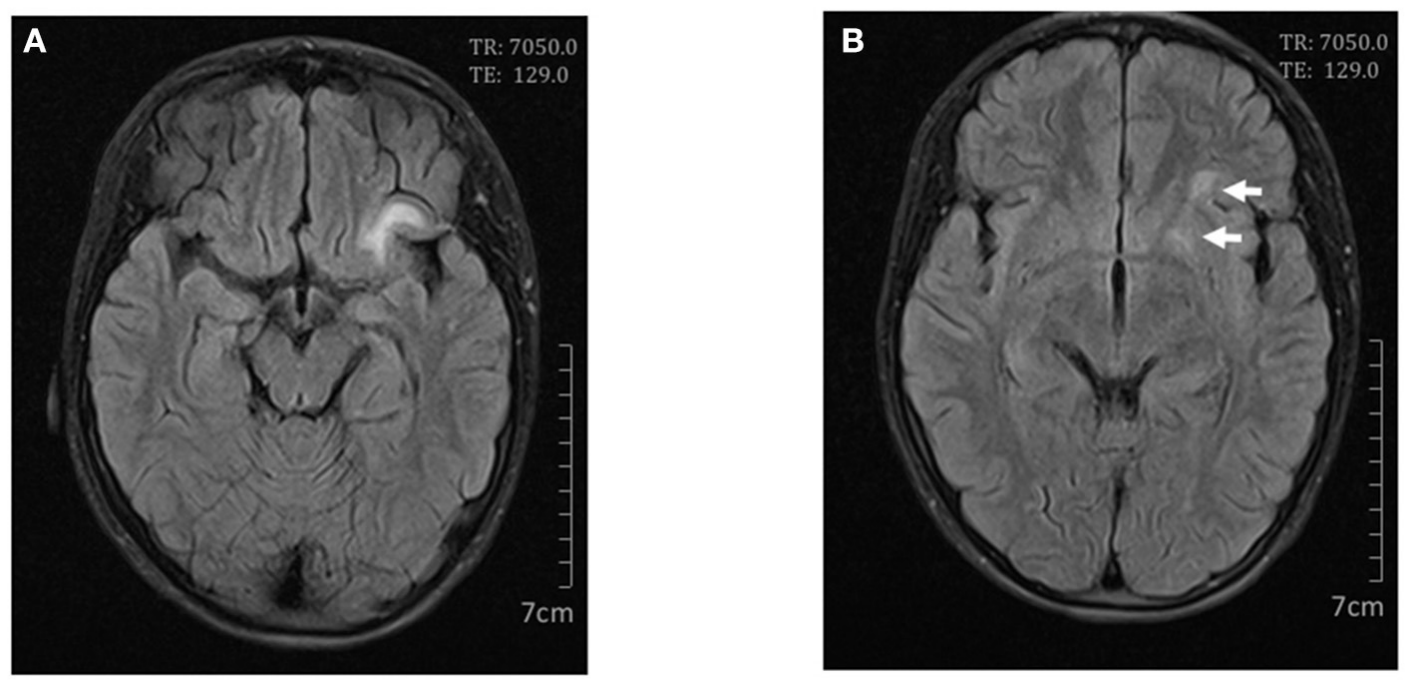
Cerebral magnetic resonance imaging on day 4. Axial FLAIR scan showed high signals in the left frontal lobe **(A)** and lentiform nucleus **(B)**.

The patient presented with fever, an ulcer on the lip, alopecia, malar rash, and rashes on the limbs. The initial laboratory results revealed leukopenia (white blood cell count 2.02 × 10^9^/L, lymphocyte count 0.70 × 10^9^/L), autoimmune hemolysis (hemoglobin 79 g/L), low C3 [0.26 g/L (normal range 0.9–1.8 g/L)], low C4 [0.04 g/L (normal range 0.1–0.4 g/L)], and antibody abnormalities. The antinuclear antibody was positive with a titer of 1:160; the anti-double-stranded DNA and anti-Smith antibodies were positive. Besides, laboratory data on admission also showed hyperferritinemia (ferritin > 1,500 μg/L), hypertriglyceridemia (4.87 mmol/L), and elevated levels of aspartate aminotransferase (123 U/L) and lactate dehydrogenase (621 U/L). The fibrinogen level (2.75 g/L) and urinalysis were normal. Cerebrospinal fluid and bone marrow examination on day 2 showed no infection or macrophages phagocytosing hematopoietic cells. Cerebral magnetic resonance imaging (MRI) was taken on day 4, revealing T2-white matte hyperintensities in her left frontal lobe and left lentiform nucleus (Figure 1). The presence of clinical and laboratory criteria led to the diagnosis of SLE and SLE-associated MAS.

The patient suffered from high fever twice a day after admission. Glucocorticoid therapy was started on day 5, with intravenous methylprednisolone 2 mg/kg/day. The next day her temperature returned to normal.

**Figure 2:**
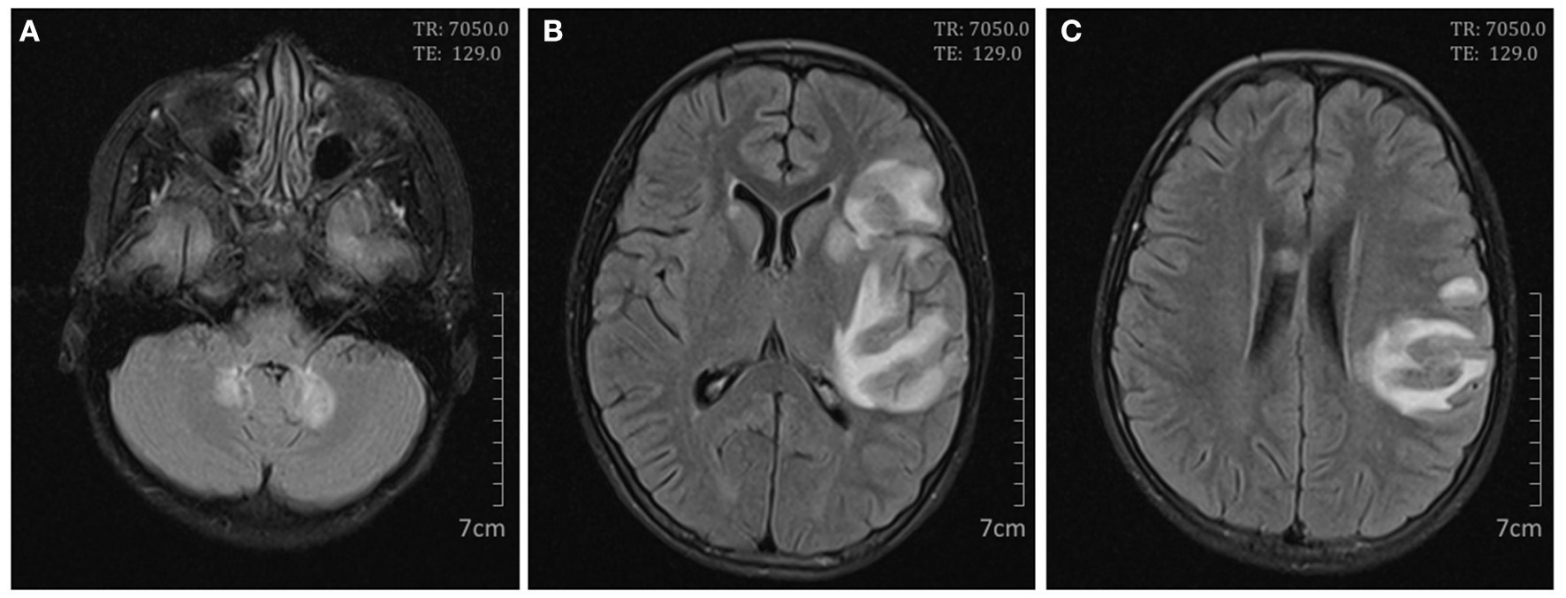
Cerebral magnetic resonance imaging on day 9. Axial FLAIR scan showed high signals in the bilateral cerebellum **(A)**; the bilateral basal ganglias and left frontal, temporal, insular, and parietal lobes **(B)**; and the right corpus callosum and left parietal lobe **(C)**.

**Figure 3:**
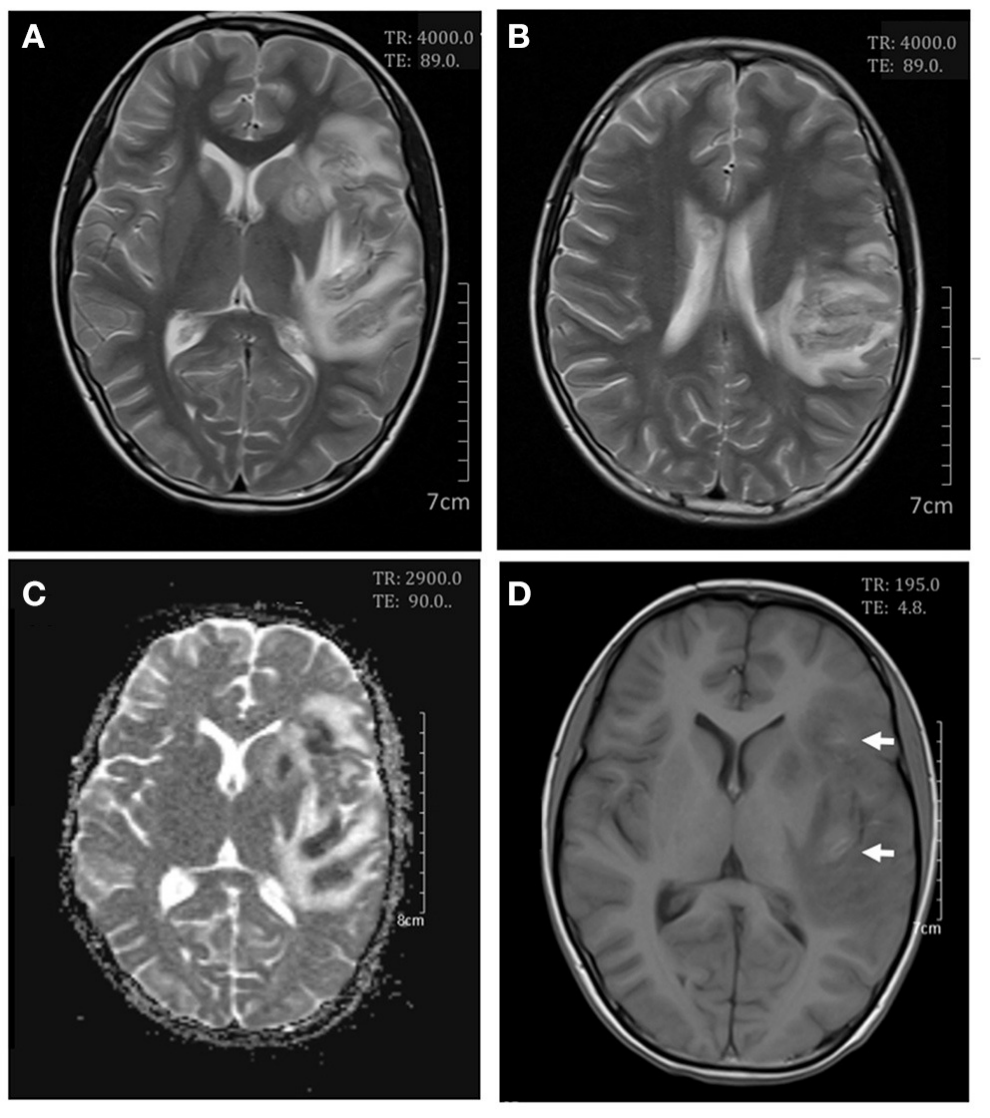
Cerebral magnetic resonance imaging on day 13. Axial T2-weighted scan showed an increase in white matter swelling **(A,B)**. Apparent diffusion coefficient maps demonstrated infarcts in the left lentiform nucleus and the frontal, temporal, insular, and parietal lobes **(C)**. Axial T1-weighted scan showed small areas of hemorrhages in the frontal and insular lobes **(D)**.

**Figure 4:**
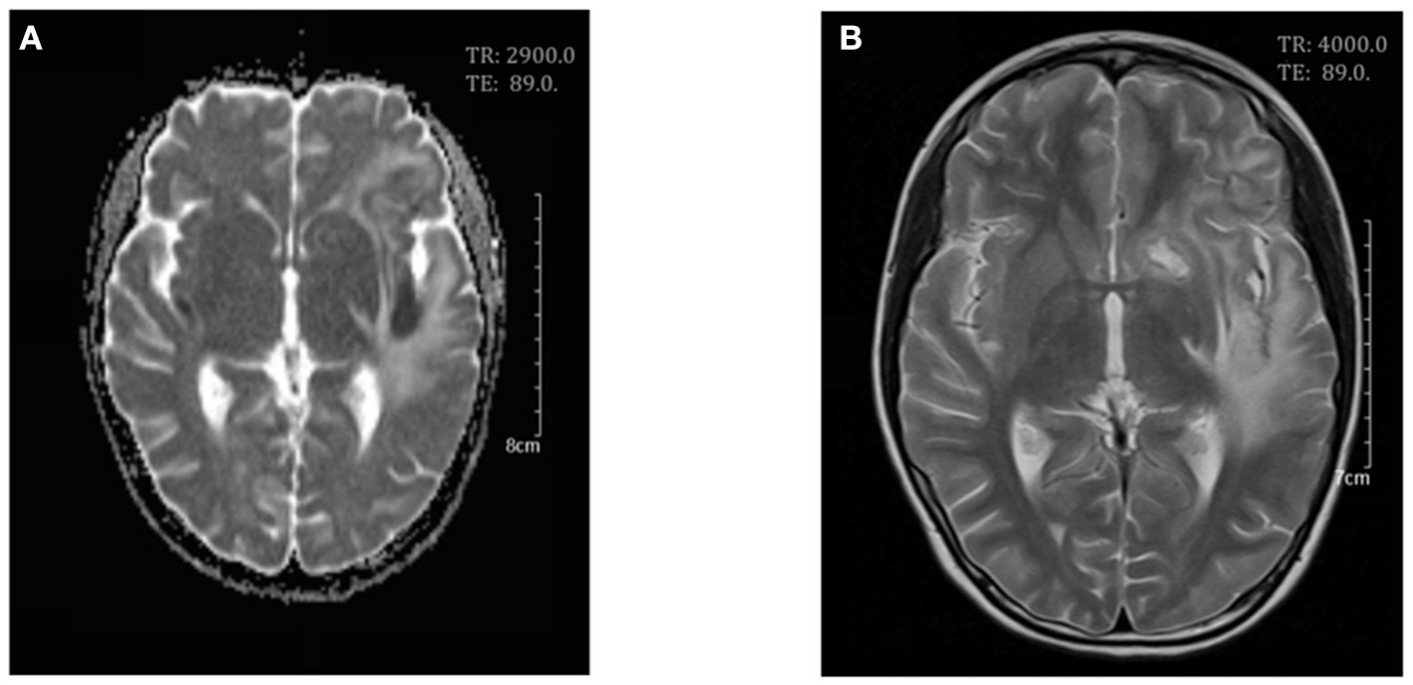
Cerebral magnetic resonance imaging on day 25. Axial T2-weighed scan **(A)** and apparent diffusion coefficient (ADC) maps **(B)** showed a decrease in white matter swelling.

On day 7 after admission the patient appeared weak and ill, then the next day she exhibited dizziness, drowsiness, apathia, and dysarthria, and her temperature reached 37.9°C. The patient was given high-dose methylprednisolone pulse therapy (30 mg/kg/day) for 3 days. On day 9 the second cerebral MRI showed rapid progress (Figure 2), with multi-focal abnormal signals mainly involving white matter (bilateral temporal, cerebellum, insular lobes, and basal ganglias; left frontal and parietal lobes; right corpus callosum). The signals were hyperintense on fluid-attenuated inversion recovery (FLAIR). Cyclophosphamide (8.5 mg/kg/day for 2 days) and intravenous immunoglobulin (2 g/kg) were initiated in the therapy. On day 10 the patient showed significant improvements in all clinical conditions, including the rashes. On day 13 the third MRI demonstrated small infarcts in the left lentiform nucleus, left frontal lobe, temporal lobe, insular lobe, parietal lobe, and right corpus callosum, revealed by decrease in apparent diffusion coefficient in the gray matter, and an increased swelling in the surrounding white matter (Figure 3). She was given intrathecal dexamethasone once a week for three times. The second pulse of high-dose methylprednisolone was administered 1 week after the first pulse. Cyclophosphamide and immunoglobulin were administered with an interval of 2 weeks. On day 25 she had a fourth MRI scan and achieved neuroimaging improvements (Figure 4). The patient was discharged on day 30, with prednisone and addition of hydroxychloroquine.

## Discussion

The actual incidence of MAS in SLE patients is still unknown because of its under-recognition; the prevalence was reported ranging from 0.9 to 9%. The mortality rate of MAS was high in patients with and without treatment (3). Early diagnosis of the MAS complication is not easy since there is no uniform diagnostic criteria, and on the other side, its similar laboratory and clinical features with the disease itself make the diagnosis more difficult (5). The HLH-2004 criteria are widely used in the diagnosis for MAS, but the sensitivity is not satisfactory; patients who meet the criteria usually have a more severe condition. In 2009, Parodi et al. set up diagnostic guidelines for MAS as complication for juvenile SLE (6). Compared with the HLH-2004 criteria, the guidelines included more variables, such as CNS dysfunction, aspartate aminotransferase, lactate dehydrogenase, and a higher sensitivity. Our patient met one clinical criterion and five laboratory criteria according to the 2009 guidelines and was diagnosed with MAS secondary to SLE (fever, cytopenia, increased aspartate aminotransferase, increased lactate dehydrogenase, hypertriglyceridemia, and hyperferritinemia).

We performed bone marrow puncture on day 2 after admission, demonstrating no hemophagocytosis. The fibrinogen level was normal on admission, but subsequent tests showed a gradual decline in fibrinogen level, which was 1.25 g/L on day 12. The absence of hemophagocytosis and lack of fibrinogen consumption are unusual in fulminant MAS. However, the absence of hemophagocytosis on biopsy does not exclude the diagnosis of MAS. Research has established that hemophagocytosis often occurs in the later stage of the disease and is only present in 32–60% of patients (3, 7). Awaiting the change on bone marrow or organ biopsy could fatally delay diagnosis and treatment. Besides, Our patient had persistent fever and a sharp rise in ferritin level, which were the best parameters to discriminate MAS from active SLE with an almost 100% sensitivity and specificity (8), and subsequent results of fibrinogen consumption supported the diagnosis of MAS. Parodi et al. pointed out that the criteria may not be powerful enough to distinguish MAS from infectious complications. We found no evidence of infection in our patient, which further points to our diagnosis of MAS.

Our patient had CNS involvement, which was found both in SLE-associated MAS and active disease with neuropsychiatric lupus (NPSLE) as its clinical spectrum. Therefore, it is difficult to distinguish between these two conditions, which is quite different in patients with systemic juvenile idiopathic arthritis, in which a CNS dysfunction is not a manifestation of the active disease. The incidence of NPSLE events has been reported ranging from 14 to 67% in SLE patients (9–12), but only 13–38% of those events could be attributable to SLE (13); the main reason is that there are no clear standards to define. To improve the attribution, some models have been proposed (14, 15) but have not been widely used in clinical practice. The prevalence of CNS involvement is 18–50% in SLE-associated MAS patients (3, 16); the attribution is also difficult because of its low incidence, and on the other side there is no specific biomarker or specific neuroimaging pattern. More than half of children with NPSLE have no conventional MRI abnormalities (17). The most common abnormalities are white matter hyperintensities, followed by brain atrophy, gray matter lesions, and basilar artery territory infarction (18). However, the neuroimaging patterns have rarely been studied in MAS patients, probably due to its low prevalence and the difficulty to distinguish it from NPSLE. In HLH patients, the most frequent changes are white matter T2 hyperintensity and encephalatrophy (19). Therefore, more research is needed in the future to meet the clinical need. In our case, we defined the CNS involvement secondary to MAS because it occurred at the time soon after the presentation of MAS and on the opinion of experts comprising two pediatric rheumatologists and one neurologist. Moreover, though there is no reliable definition, most experts of HLH agree that an abnormal MRI of the brain defines CNS-HLH (20).

The neuroimaging of our patient showed widespread lesions, involving multi-lobes, which was rarely reported in MAS patients. We found three cases of MAS reporting CNS imaging but no case of SLE-associated MAS in the literature (21–23). Our patient presented multiple T2-hyperintense area mainly in the white matter, as well as small infarcts and micro-bleedings in the gray matter. The CNS abnormalities result from activated lymphocytes and macrophages infiltration in leptomeninges and brain parenchyma with multifocal necrosis and reactive astrogliosis. The patient had persistent high fever after admission, and a cerebral MRI was taken on day 4, revealing abnormal signals. Given the first MRI result, we were alert to severe disease progression when she developed CNS symptoms. Patients of MAS with CNS involvements were associated with a higher mortality rate (9). High dose of methylprednisolone, cyclophosphamide, and immunoglobulin were initiated. A rapid and complete recovery of CNS manifestations was achieved in the patient, proving our prompt recognition and management.

The present case reports a child of SLE with MAS and multi-focal lesions on cerebral MRI as the onset presentations and suggests that we should have a great awareness of SLE-associated MAS. Early cerebral MRI examination of disease evaluation may be recommended in SLE-associated MAS patients for early recognition and treatment of severe cases.

## Data Availability Statement

The original contributions presented in the study are included in the article/supplementary material, further inquiries can be directed to the corresponding author.

## Ethics Statement

The studies involving human participants were reviewed and approved by the Ethics Committee of the Children' Hospital, Zhejiang University School of Medicine (2020-IRB-025). Written informed consent to participate in this study was provided by the participants' legal guardian/next of kin.

## Author Contributions

ML designed the work and revised the manuscript. NS and XW carried out the study and prepared the manuscript. XY performed and analyzed the magnetic resonance imaging. LZ and QM provided the clinical data. All authors contributed to the article and approved the submitted version.

## Funding

The authors received financial support from Zhejiang Basic Public Welfare Research Project (LGF19H100002).

## Conflict of Interest

The authors declare that the research was conducted in the absence of any commercial or financial relationships that could be construed as a potential conflict of interest.

## Publisher's Note

All claims expressed in this article are solely those of the authors and do not necessarily represent those of their affiliated organizations, or those of the publisher, the editors and the reviewers. Any product that may be evaluated in this article, or claim that may be made by its manufacturer, is not guaranteed or endorsed by the publisher.

## Abbreviations

CNS: central nervous system
FLAIR: fluid-attenuated inversion recovery
HLH: hemophagocytic lymphohistiocytosis
MAS: macrophage activation syndrome
MRI: magnetic resonance imaging
SLE: systemic lupus erythematosus.
